# Factors Affecting the Quality of Sleep in Children

**DOI:** 10.3390/children8060499

**Published:** 2021-06-12

**Authors:** Jun Kohyama

**Affiliations:** Department of Paediatrics, Tokyo Bay Urayasu Ichikawa Medical Centre, Toudaijima 3-4-32, Urayasu 279-0001, Japan; info@j-kohyama.jp

Sleep quality is difficult to define objectively. Even if the polysomnographic recording of a person showed a typical sleep progress chart of a night with a higher rate of deep sleep in the first third of the night, an increasing REM sleep and N2 duration in the last third of the night, and a low incidence of intermittent waking, the quality of the night’s sleep would be defined as poor if he/she was unsatisfied with the night’s sleep. So far, we have to define the quality of sleep subjectively. On the other hand, the need for sleep quantity has individual variabilities, which are influenced by genetic, behavioral, medical, and environmental factors. Moreover, sleepiness was recently reported to be a stronger predictor of academic performance (one of the important aspects of daytime brain functioning) than the quantity of sleep. Thus, many researchers have focused on searching for ways to assess the quality of sleep. From a similar point of view, restlessness and/or restfulness could also be a candidate that reflects on sleep quality. The goal of this Special Issue in Children was to highlight recent data in the context of children’s sleep quality. Finally, we succeeded in publishing seven high-quality papers.

Fadzil [1] defined the adequate quality of sleep as one’s “feeling fresh” after waking up and listed the following five issues as factors affecting the quality of a child’s sleep; 1. gene, 2. parent/caregiver, 3. sleep disorders and medical problem, 4. sleep habits/environment and medications, and 5. screen exposure.

Galan–Lopez et al. [2] assessed both the quality and quantity of sleep in 13- to 16-year-old European adolescents (*n* = 1717) living in three different European countries (Spain, Iceland and Estonia). They used the Pittsburgh Sleep Quality Index (PSQI) to score the sleep quality. Although they did not find sleep quantity differences among the three countries, they found that the female gender and adolescents in Spain and Estonia had a poor sleep quality. They mentioned the effects of cultural differences on both sleep quality and quantity, and described school start times, extracurricular school sports, homework, part-time work, lack of parental limit-setting around bedtimes, and diet or body fat percentage as those factors.

Sawa et al. [3] investigated associations of lifestyle and familial and social factors with sleep habits in 1882 elementary school children. They found that a night-oriented lifestyle was associated with a poor sleep habit. In terms of daytime sleepiness, physical inactivity was found to be a significant associated factor. They also found that children in the 5th and 6th grades (aged 10–13 years) were significantly more likely to experience daytime sleepiness compared with 1st graders (aged 6–7). A short sleep duration was concluded to be a predictor of daytime sleepiness in these grades.

The following three studies focused on the COVID-19-related school closure. Shinomiya et al. [4] assessed the effects of COVID-19-related nursery school closures on sleep and the general behavior of infants and parents. In infants, the total sleep time and percentage of outdoor play decreased significantly, and media use increased significantly. The percentage of caregivers responding with “negative childcare feelings” was significantly higher in the group with fewer than three nursery school attendance days per week. They emphasized the role of physical activity and media use on sleep and stress.

Komada et al. [5] also assessed changes of sleepiness in elementary school children during a temporary school closure due to COVID-19. They used the Japanese version of the Pediatric Daytime Sleepiness Scale (PDSS-J) and found that a COVID-19-related school closure decreased sleepiness in children. They found four items (advancing wakeup time on weekends, advancing midsleep on weekends, decreasing total sleep time on weekends, and decreasing social jetlag) that were significant factors associated with decreasing sleepiness scores.

Nakayama et al. [6] investigated the effects of COVID-19-related school closures on lifestyle and internet use among 2270 junior high school students. They found that the average score of the Japanese version of Young’s Diagnostic Questionnaire, which can assess addiction, and the rate of problematic internet users in a survey conducted just after COVD-19-related school closures increased and were higher than those in the other surveys conducted on June 2018 and 2019, but the differences were not statistically significant.

Since it remains to be investigated whether dietary intake affects sleep duration and quality, Lee et al. [7] assessed, as a first step, the association between nutrient intake and sleep duration among 1422 adolescents aged 12–18 years. They found that a higher intake of fiber and lower intake of sodium were associated with a longer sleep duration. Further studies on the association between dietary intake and sleep quality are expected.

I believe this special issue contributes to the enhancement of further studies on sleep quality among children and adolescents.
